# Supplementary material for: A new design of wind power prediction method based on multi-interaction optimization informer model
Source: PLoS One. 2025 Aug 28;20(8):e0330464. doi: 10.1371/journal.pone.0330464 (PMC12393750; doi:10.1371/journal.pone.0330464)
Supplement: Attachment — Submitted filename: Review-PONE-D-25-26282.pdf [file pone.0330464.s001.pdf]

“A New Design of Wind Power Prediction Method Based on Multi-Interaction Optimization Informer Model”

Paper: PONE-D-25-26282

This manuscript presents a new wind power prediction framework named MFIO-Informer, which enhances the conventional Informer model through a multi-source feature interaction mechanism and health-status-aware modeling. The authors employed Lasso and Pearson correlation for feature selection, a feedforward neural network (FNN) for extracting dynamic synergy coefficients, and a novel health matrix for optimizing the encoder, decoder, and embedding process of the Informer. Experimental validation was conducted using two publicly available datasets.

In summary, the research is technically robust, with a detailed methodology and well-documented performance improvements. The article effectively tackles a critical and practical problem in renewable energy forecasting. The findings are valuable for both researchers and industry professionals. However, several issues and concerns must be resolved, so I recommend that the paper undergo a revision before acceptance. The following points should be considered in the revised version:

Problems:

1. Clarify how the health matrix A is computed in practice.

2. Have you tested the MFIO-Informer in an online or real-time prediction setting, or is the evaluation entirely offline?

3. Perform a sensitivity analysis on the number of encoder/decoder layers, embedding dimensions, or DSC inclusion.

4. Were the model parameters and hyperparameters selected manually with trial and error or via automated optimization techniques? Provide the related tables. If previous research is used, appropriate referencing should be provided.

5. Add diagrams comparing the MFIO-Informer architecture with the standard Informer for better visual clarity.

6. Expand the related work section and add comparisons with non-Informer baselines.

7. Clarify mathematical formulations in the health matrix section with a step-by-step example

8. The convergence graph of the final network should be presented in terms of epochs.

9. The performance results of the proposed model should be compared with the models of other researchers and reported in a table.

10. The execution time and training time of the proposed model should be compared with those of other researchers.

11. The paper has several English problems. Correct them.
